# Supplementary material for: The interactome of intact mitochondria by cross-linking mass spectrometry provides evidence for coexisting respiratory supercomplexes
Source: Mol Cell Proteomics. 2017 Dec 8;17(2):216–32. doi: 10.1074/mcp.RA117.000470 (PMC5795388; doi:10.1074/mcp.RA117.000470)
Supplement: Supplemental Data [file supp_RA117.000470_133922_0_supp_23978_fzffwf.zip › spectra_annotation/mito_DR_spectra_annotation/375-1-9-1-6-1.pdf]

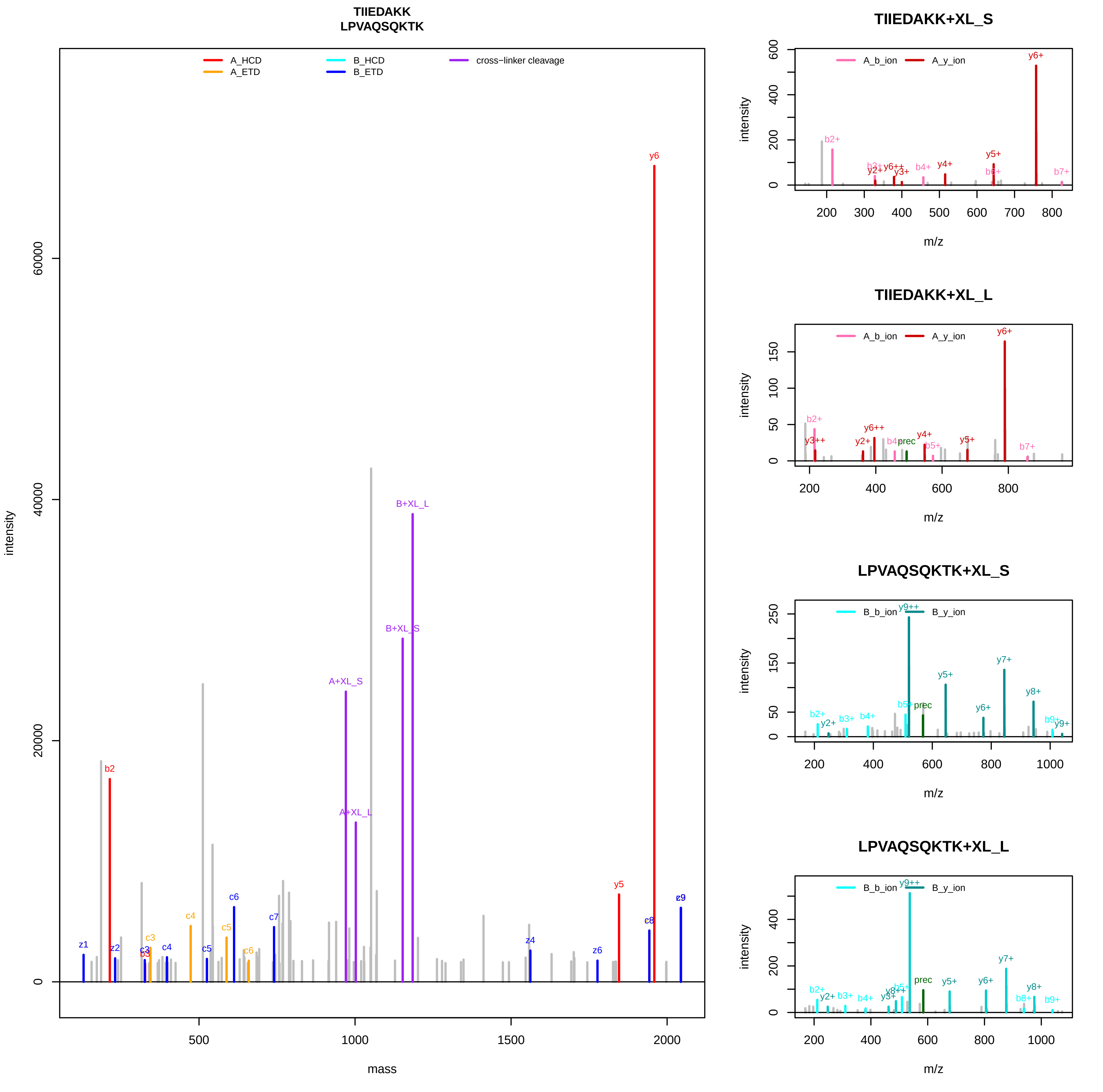

TIIEDAKK
LPVAQSQKTK
A_HCD
A_ETD
B_HCD
B_ETD
cross−linker cleavage
intensity
mass
y6
B+XL_L
B+XL_S
A+XL_S
A+XL_L
b2
y5
c8
z9
z4
z6
c6
c7
c4
c5
c3
c6
z1
z2
c3
b3
c4
c5
TIIEDAKK+XL_S
TIIEDAKK+XL_L
LPVAQSQKTK+XL_S
LPVAQSQKTK+XL_L
A_b_ion
A_y_ion
B_b_ion
B_y_ion
m/z
prec
y9++
y7+
y8+
y6+
y5+
b2+
y2+
b3+
b4+
b5+
b9+
y9+
y6+
y5+
y4+
y3+
y2+
y6++
b7+
